# Wenckebach Block due to Hyperkalemia: A Case Report

**DOI:** 10.1155/2010/879751

**Published:** 2011-01-23

**Authors:** Aparajita Sohoni, Berenice Perez, Amandeep Singh

**Affiliations:** Department of Emergency Medicine, Alameda County Medical Center, Highland Hospital, Highland Campus, 1411 East 31st Street, Oakland, CA 94602, USA

## Abstract

Hyperkalemia is a commonly encountered electrolyte abnormality that can significantly alter normal cardiac conduction. Potentially lethal dysrhythmias associated with hyperkalemia include complete heart block and Mobitz Type II second-degree AV block. We report a unique case of Mobitz Type 1 second-degree atrioventricular (AV) block, known commonly as Wenckebach, due to hyperkalemia. The patient's symptoms and electrocardiogram (ECG) evidence of Wenckebach block resolved with lowering of serum potassium levels, with subsequent ECG showing first-degree AV block. This paper highlights an infrequently reported dysrhythmia associated with hyperkalemia that emergency physicians should be familiar with.

## 1. Introduction

Hyperkalemia is a common and potentially life-threatening electrolyte abnormality [1]. While the incidence of hyperkalemia in the general population is not known, it is approximated that this electrolyte disturbance occurs in 1–10% of hospitalized patients annually and carries a mortality rate of 1 per 1,000 patients [1]. Hyperkalemia is associated with significant disturbances in cardiac conduction, ranging from QT interval shortening, to PR interval lengthening and QRS widening [2]. Reversible fascicular blocks, as well as bundle branch blocks or intraventricular conduction delay can be seen. Moreover, hyperkalemia is known to cause potentially lethal dysrhythmias including ventricular tachycardia, ventricular fibrillation, idioventricular rhythms, and asystole [1–9]. 

Despite the range of heart blocks associated with hyperkalemia, Mobitz Type 1 second-degree AV block or Wenckebach is infrequently reported in the medical literature. Hyperkalemia-induced Wenckebach in the setting of a pacemaker, termed pacemaker exit block, has been previously reported [9]. In the absence of a pacemaker, there is only a single reported case of hyperkalemia-induced Wenckebach—however, this case occurred in a patient with significant pre-existing underlying cardiac disease [4]. To our knowledge, there is no report of hyperkalemia-induced Wenckebach in the absence of pre-existing documented cardiac disease, conduction abnormality, or pacemaker. We present a case of symptomatic hyperkalemia presenting as Wenckebach, with resolution of this form of AV block as serum potassium levels were corrected. 

## 2. Case Report

A 68-year-old man was brought in by ambulance after his wife called EMS upon finding her husband sitting on the toilet and complaining of head and neck pain. Per the paramedic report, the patient was found seated on the toilet, cool, pale, diaphoretic, and confused. He had no palpable radial pulses. He was found to have a heart rate of 30 beats per minute (bpm) on the field cardiac monitor. He received 0.5 mg of atropine intravenously (IV) in the field with some improvement in his heart rate and mental status and was then paced transcutaneously to a heart rate of 80 bpm. EMS also placed him on 15 liters of oxygen by non-rebreather (NRB) mask, documented a blood glucose level of 499 mg/dL, and initiated an intravenous (IV) bolus of 500 mL of normal saline. Repeat vital signs immediately prior to arrival in the Emergency Department (ED) were notable for a systolic blood pressure of 90 mmHg, pulse 80 bpm, respirations 18 breaths per minute, and pulse oximetry of 100% on 15 L NRB mask. His cardiac rhythm on the monitor demonstrated a paced rhythm at 80 bpm without ectopy, and he was transported without any further event. 

Upon arrival to the ED, his mental status had improved. He was awake and answered questions appropriately. He reported a history of diabetes mellitus type II, hypertension, hyperlipidemia, chronic renal insufficiency, and prostate cancer. He described one hospitalization several months prior, in Mexico, for hyperkalemia. He did not know the details of that hospitalization but denied ever undergoing hemodialysis. His medications were listed as atenolol, diltiazem, hydrochlorothiazide, losartan, and metformin. He denied any recent medication changes, overdoses, or chest pain. He had not taken his medications that morning. 

The external pacer was stopped, and he was found to have a heart rate of 45 bpm and a manual blood pressure of 90/50 mmHg. His physical examination was remarkable only for bradycardia and cool lower extremities with a delayed capillary refill. He did not have any increased work of breathing, abnormal breath sounds, irregular heart sounds, jugular venous distention, or lower extremity edema. 

**Figure 1:**
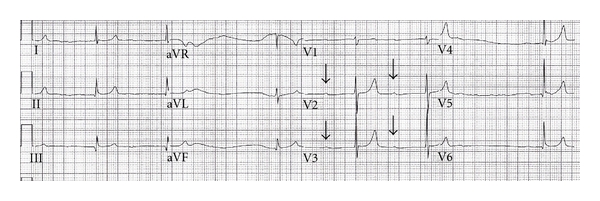
Initial ECG demonstrating tall, symmetric T waves, and prolonged PR intervals (P waves marked with arrows).

**Figure 2:**
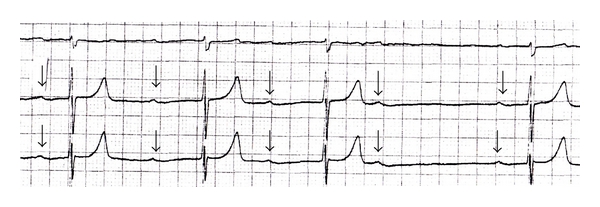
Rhythm strip showing progressive PR prolongation with subsequent dropped QRS complex, consistent with Wenckebach (P waves marked with arrows).

His initial ECG showed a calculated ventricular rate of 36 bpm and a QRS duration of 76 milliseconds (ms). The providers further interpreted this ECG to show a prolonged PR interval and peaked T waves (Figure 1). His rhythm strip revealed grouped beating, with a PR interval that progressively prolonged with each of three successive QRS complexes, followed by a nonconducting P wave (Figure 2). Given these findings, the rhythm was interpreted as type I second-degree AV block, or Wenckebach. 

A rapid serum electrolyte level was obtained using an i-Stat device. This showed serum potassium level of 8.0 mmol/L [3.6–5.0]. His serum chemistries were received shortly thereafter and were significant for sodium 130 mmol/L [137–145], potassium 8.3 mmol/L, chloride 102 mmol/L [98–107], CO2 21 mmol/L [22–30], BUN 28 mg/dL [7–21], creatinine 1.7 mg/dL [0.5–1.4], glucose 528 mg/dL [65–110], magnesium 1.6 mg/dl [1.6–2.3], and phosphorus 6.0 mg/dL [2.5–4.5]. 

The patient's hyperkalemia was treated with calcium gluconate, insulin, kayexalate, and albuterol nebulizer treatments. He was also given an IV bolus of two liters of normal saline. Four hours later, his serum potassium level was 6.0 mmol/L. 

**Figure 3:**
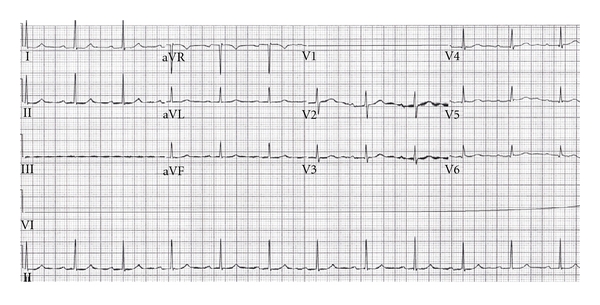
ECG following resolution of hyperkalemia, with first-degree AV block (PR interval 232 ms) and resolution of peaked T waves.

The patient was admitted to the internal medicine service and nephrology was consulted. His hyperkalemia was thought to be secondary to renal tubular acidosis type 4. His cardiac enzymes were negative for ischemia. At the time of discharge his potassium level was 3.9 mmol/L and his creatinine was 1.1 mg/dL. The patient's beta-blocker and calcium-channel blocker medications were held throughout his hospital stay. His ECG immediately prior to discharge demonstrated resolution of the previously seen Wenckebach pattern, however, with a persistently prolonged PR interval of 232 ms consistent with type 1 AV block, and normal T wave morphology (Figure 3). His discharge medications included lantus, diltiazem, kayexalate, and pravachol.

## 3. Discussion

This case demonstrates an atypical presentation of hyperkalemia-induced Wenckebach that resolved to first-degree AV block with lowering of the serum potassium levels. Although electrophysiologic studies at the AV node and His-Purkinje system would be needed to confirm our theory that hyperkalemia induced the ECG pattern of Wenckebach in this patient, the absence of any other explanation, such as medication overdose, myopericarditis, rheumatic fever, or acute myocardial ischemia, makes it seem very unlikely that this was the case. The resolution of Wenckebach to first-degree AV block with aggressive treatment of hyperkalemia also suggests that this electrolyte disturbance produced the higher-grade AV block. 

The AV node is known to be susceptible to hyperkalemia, producing the classic prolonged PR interval and QRS widening seen often in the setting of hyperkalemia. The development of advanced heart blocks (second- and third-degree AV blocks) is generally found only in patients with pre-existing heart failure, conduction abnormalities, or other cardiac disease [4]. Pre-existing cardiac disease has long been thought to potentiate the cardiac-conduction blocking effects of potassium, especially with the generation of second- and third-degree heart blocks [2, 4, 6]. This effect was postulated to occur because underlying coronary artery disease or diffuse cardiac disease was thought to have already damaged the conduction path through the AV node and His-Purkinje system, and that an elevated potassium level only further worsened this pre-existing conduction abnormality. 

In our patient, in the absence of acute myocardial infarction, we propose that Wenckebach resulted due to the patient's AV node's susceptibility to hyperkalemia, which was likely heightened by the patient's use of both a beta-blocker and a calcium-channel-blocker (atenolol and diltiazem), as well as likely underlying AV node disease evidenced by his final ECG showing first-degree block. Hyperkalemia likely contributed to dampening the pacemaker function of the AV node and worsening underlying conduction disease, thereby producing Wenckebach. 

The previous documented cases of hyperkalemia-associated Wenckebach are interesting in that all cases occurred in settings of severe pre-existing cardiac disease. In the most dramatic cases, those of Wenckebach pacemaker exit block due to hyperkalemia, elevated potassium levels cause progressive latency in the generation of a QRS complex from the time of the pacemaker spike [9]. Hyperkalemia causing decreased intraventricular conduction, manifested by prolongation of the His-Ventricle (HV) interval on EP studies, and widening of the QRS interval on ECG, results in failure of the pacemaker stimulus to propagate and a prolonged ventricular refractory period [9]. The majority of cases of Wenckebach pacemaker exit block have grim prognoses [9]. 

The only other case of hyperkalemia-induced Wenckebach is reported in an 87-year-old female with a history of hypertension and congestive heart failure, whose baseline ECG demonstrated a right bundle branch block and a left anterior fascicular block [4]. In the setting of hyperkalemia (K + 7.6 mmol/L), her ECG progressed to show Wenckebach [4]. EP studies done at during the episode of hyperkalemia localized the conduction delay to the AV node, with no significant worsening of conduction in the His-Purkinje system [4].

## 4. Conclusion

Hyperkalemia is a commonly encountered electrolyte abnormality that can produce life-threatening derangements in cardiac conduction. The ED physician should be aware of the range of dysrhythmias attributed to hyperkalemia—including Wenckebach and progressively higher grades of AV block—and should institute treatment rapidly to minimize patient morbidity and mortality.

##  Conflicts of Interest

None of the authors of this paper have any financial or other conflicts of interest related to this submission.
